# Supplementary material for: Altered structural balance of resting-state networks in autism
Source: Sci Rep. 2021 Jan 21;11:1966. doi: 10.1038/s41598-020-80330-0 (PMC7820028; doi:10.1038/s41598-020-80330-0)
Supplement: Supplementary file 1 — Supplementary material 1 [file 41598_2020_80330_MOESM1_ESM.pdf]

Supplementary Materials

Altered structural balance of resting-state networks in autism

Z. Moradimanesh[1], R. Khosrowabadi[1], M. Eshaghi Gordji[1,2], and G. R. Jafari[3,1,4,*]

[1]Institute for Cognitive and Brain Sciences, Shahid Beheshti University, G.C., Evin, Tehran 19839, Iran

[2]Department of Mathematics, Semnan University, P.O. Box 35195-363, Semnan, Iran

[3]Department of Physics, Shahid Beheshti University, G.C., Evin, Tehran 19839, Iran

[4]Department of Network and Data Science, Central European University, Nador u. 9, H-1051 Budapest, Hungary

[*]g_jafari@sbu.ac.ir

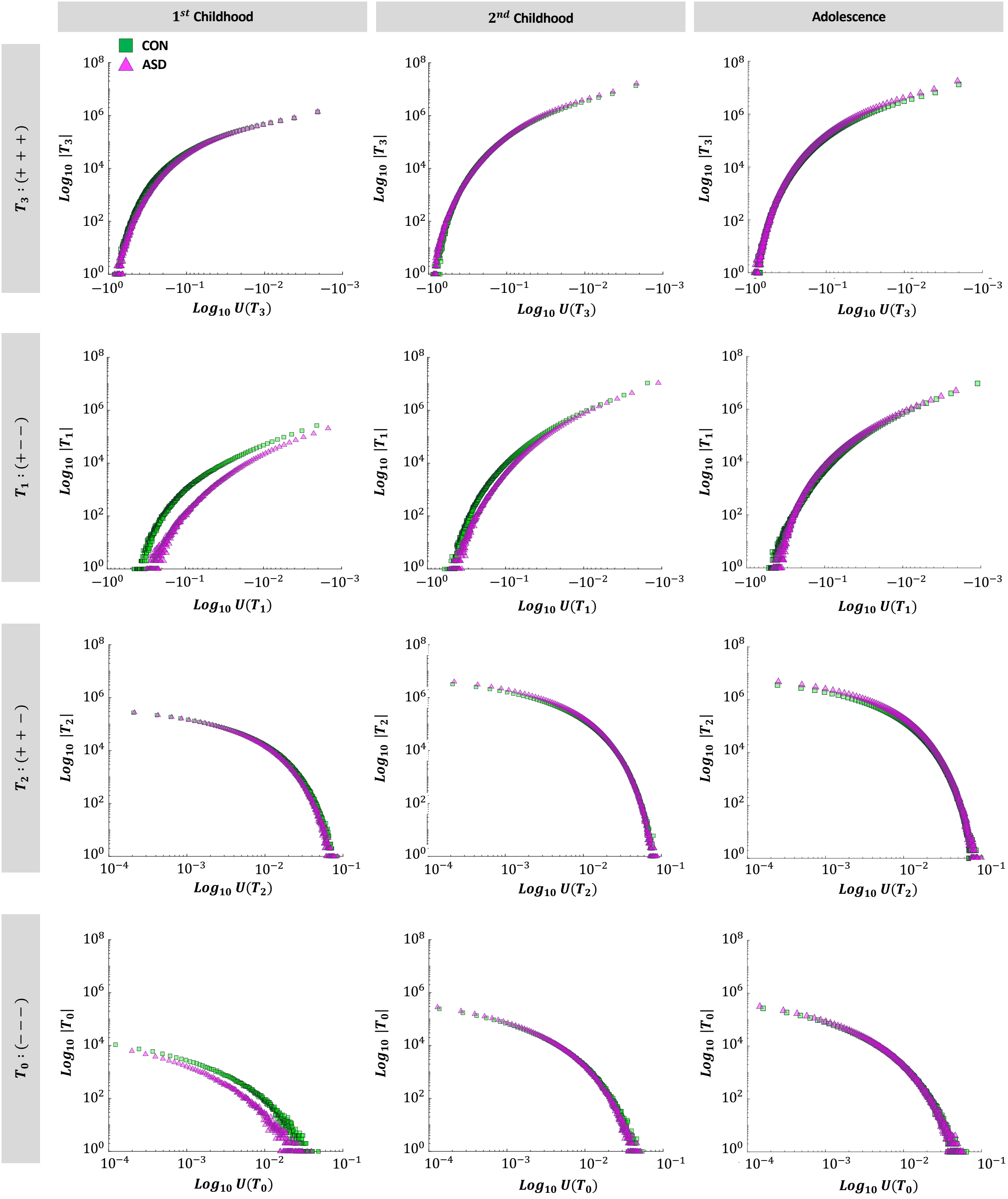

Supplementary figure S 1: The energy distributions of different types of triads during development. The energy distributions of ASD group (purple/ dark triangles) lag behind CON group (green/ light squares) for T_1 and T_0 triads during 1st childhood. $|T_i|$ = Total number of T_i, $U(T_i)$ = The energy of T_i, CON = Control, ASD = Autism spectrum disorder (Color Online).

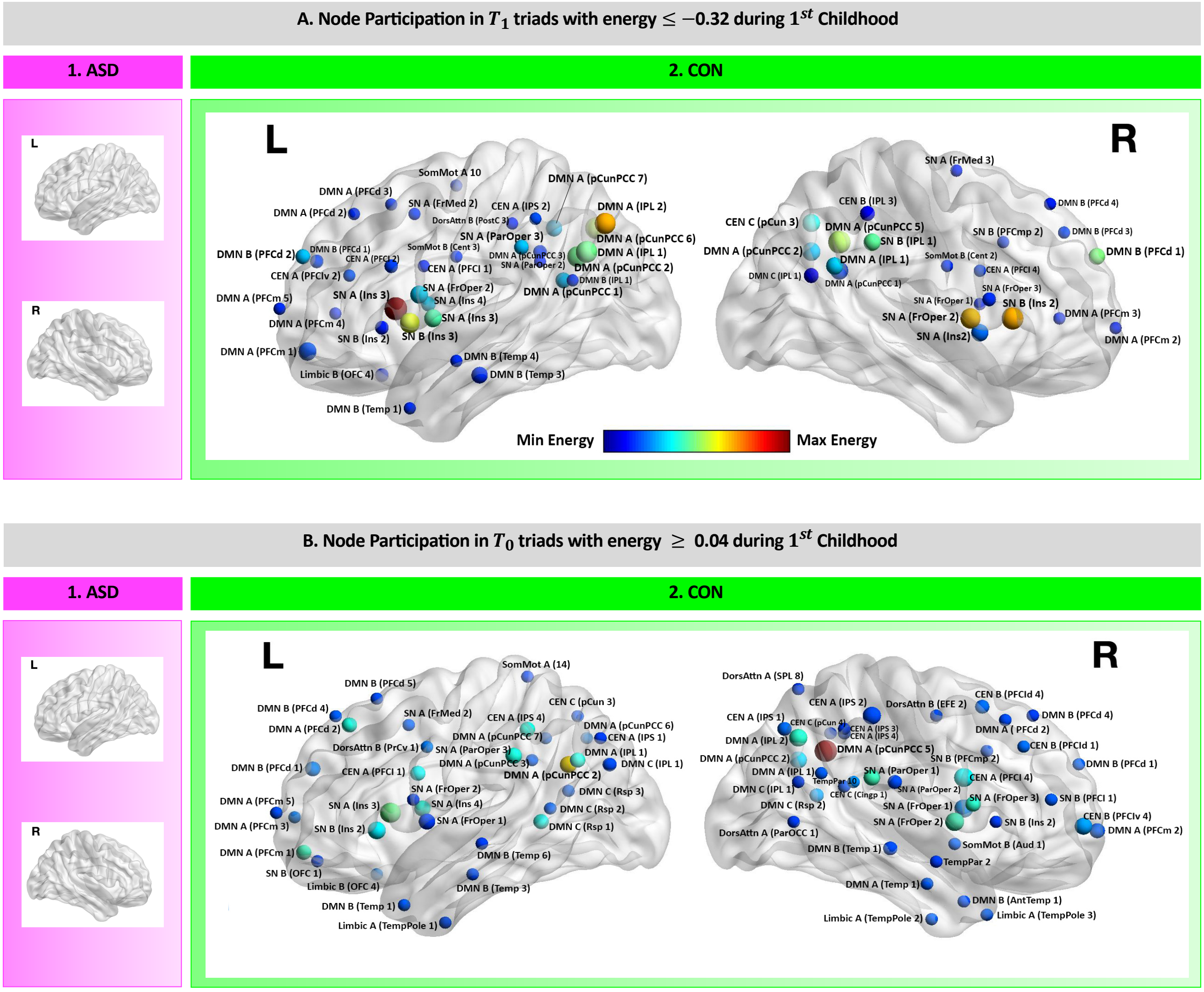

Supplementary figure S 2: Node participation in high energy T_1 and T_0 triads during 1st childhood. A. The ASD brain network (A.1) is empty above an energy threshold, here $U(T_1) \leq -0.32$, yet in the CON network (A.2) many regions from different functional sub-networks participate so the final network have high energy T_1 triads. **B.** The same pattern holds for T_0 triads. DMN = The default mode network, SN = The salience / ventral attention network, CEN = The central executive network, SomMot = The somato motor network, DorsAttn = The dorsal attention network, TempPar = The temporo-parietal network, Ins = The insula, PFC = The prefrontal cortex, Temp = The temporal cortex, FrOper = The frontal opercular, ParOper = The parietal opercular, FrMed = The frontal medial, OFC = The orbitofrontal cortex, PCC = The posterior cingulate cortex, pCun = The precuneus, IPL = The inferior parietal lobule, RSP = The retrosplenial cortex, CON = Control, ASD = Autism spectrum disorder (Color Online).

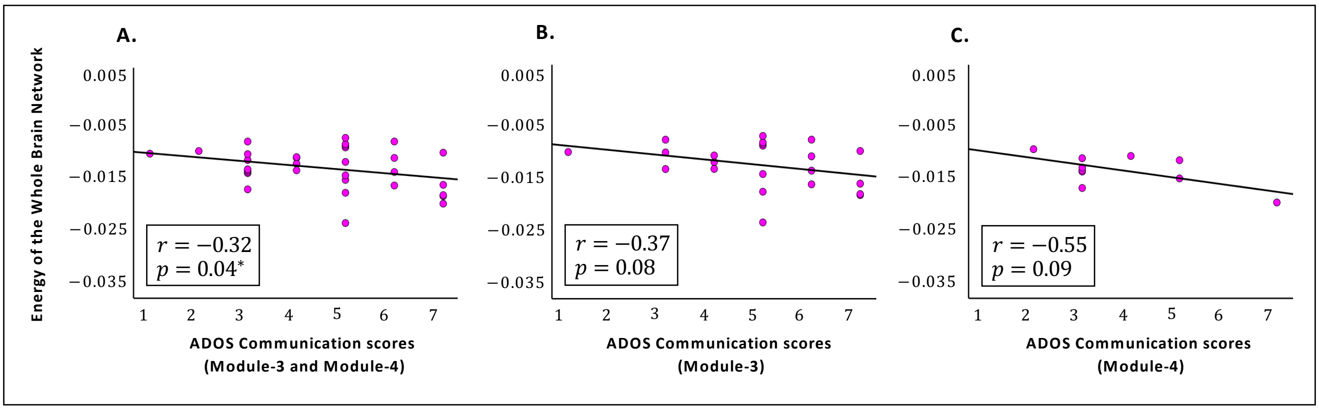

Supplementary figure S 3: Correlations between energy of the whole brian network and ADOS communication scores during adolescence. A. The Pearson's correlation is significant when considering both modules. **B, C.** However, this correlation is not significant when taking into account single module per analysis, possibly due to small samples. Thus, further investigations are encouraged. ADOS = The autism diagnostic observation schedule.

		NYU	SDSD	UM-1	UM-2	USM	Yale	Total
CON	1st Childhood	14	1	1	–	1	3	20
	2nd Childhood	22	–	17	–	4	9	52
	Adolescence	16	5	10	15	8	4	58
ASD	1st Childhood	12	–	2	–	–	4	18
	2nd Childhood	25	–	18	1	1	7	52
	Adolescence	8	1	12	11	23	3	58
Total		97	7	60	27	37	30	258

Supplementary table S 1: Number of participants per site. After applying inclusion criterion and preprocessing steps total number of 258 individuals have been remained, each from one of the five sites in the ABIDE I dataset as depicted here. NYU = New York University Langone Medical Center, SDSU = San Diego State University, UM-1 / UM-2 = University of Michigan, USM = University of Utah School of Medicine, YALE = Yale Child Study Center, CON = Control, ASD = Autism spectrum disorder.

Site	NYU	SDSU	UM	USM	Yale
Scanner	Siemens 3T Allegra	GE 3T MR 750	GE 3T Signa	Siemens 3T Trio Tim	Siemens 3T Trio Tim
TR / TE (ms)	2000/15	2000/30	2000/30	2000/28	2000/25
Flip Angle	90	90	90	90	60
Resolution (mm)	3×3×4	3.4×3.4×3.4	3.4×3.4×3	3.4×3.4×3	3.4×3.4×4
Volumes	180	180	300	240	200
Matrix	80×80×33	64×64×42	64×64×40	64×64×40	64×64×34
Slice order	Interleaved Ascending	Interleaved Ascending	Sequential Ascending	Interleaved Ascending	Interleaved Ascending

Supplementary table S 2: Functional scan parameters of different sites. TR = The repetition time, TE = The echo time, NYU = New York University Langone Medical Center, SDSU = San Diego State University, UM = University of Michigan, USM = University of Utah School of Medicine, YALE = Yale Child Study Center.
